# Self-Organizing Neural Networks Based on OxRAM Devices under a Fully Unsupervised Training Scheme

**DOI:** 10.3390/ma12213482

**Published:** 2019-10-24

**Authors:** Marta Pedró, Javier Martín-Martínez, Marcos Maestro-Izquierdo, Rosana Rodríguez, Montserrat Nafría

**Affiliations:** 1 Departament d’Enginyeria Electrònica, Universitat Autònoma de Barcelona, 08193 Bellaterra, Spain; javier.martin.martinez@uab.es (J.M.-M.); rosana.rodriguez@uab.es (R.R.); montse.nafria@uab.es (M.N.); 2 Institut de Microelectrònica de Barcelona, IMB-CNM, CSIC, 08193 Barcelona, Spain; marcos.maestro@imb-cnm.csic.es

**Keywords:** memristors, neuromorphic engineering, OxRAM, self-organization maps, synaptic device

## Abstract

A fully-unsupervised learning algorithm for reaching self-organization in neuromorphic architectures is provided in this work. We experimentally demonstrate spike-timing dependent plasticity (STDP) in Oxide-based Resistive Random Access Memory (OxRAM) devices, and propose a set of waveforms in order to induce symmetric conductivity changes. An empirical model is used to describe the observed plasticity. A neuromorphic system based on the tested devices is simulated, where the developed learning algorithm is tested, involving STDP as the local learning rule. The design of the system and learning scheme permits to concatenate multiple neuromorphic layers, where autonomous hierarchical computing can be performed.

## 1. Introduction

**Figure 1:**
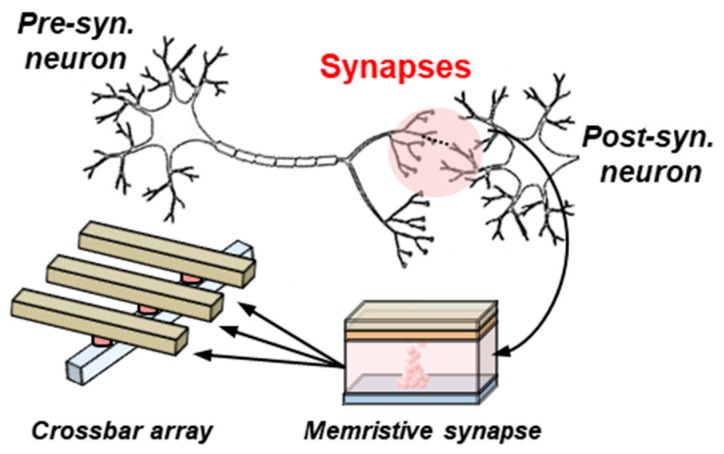
Electronic synapses can be implemented with memristive devices. The electronic neural network is implemented in a synaptic matrix layer.

**Figure 2:**
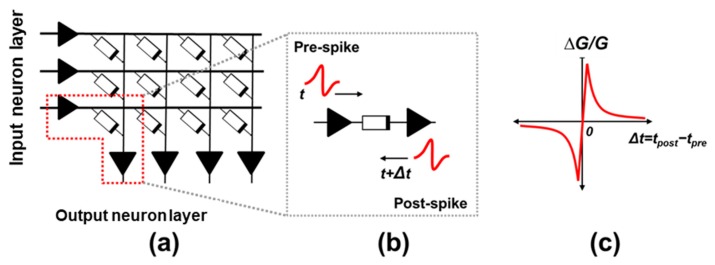
(**a**) Neuromorphic memristive array. Each node within the crossbar corresponds to the weighted connection (synapse) between two neurons, implemented with a memristor. (**b**) The conductivity of the device can be changed according to the activity of the neurons it connects. (**c**) STDP function.

The implementation of electronic synapses is nowadays one of the challenges of hardware-based neuromorphic engineering, which aims to design electronic circuits with a similar architecture and behavior to the one found in biological brains. Within this context, the conductivity of an electronic device with memristive characteristics is identified as the weight or strength of a connection between two neurons (Figure 1), usually within a crossbar array which implements the synaptic matrix layer of an electronic neural network (Figure 2a). An analog behavior of the electronic synapse is desirable to improve the robustness of the network [1,2,3], showing a large window between its higher and lower conductivities and displaying many accessible conductivity levels in between. The conductivity of an analog synaptic device is then finely tuned according to some learning rule during the training stage of a learning algorithm. Among the different technologies that have been proved to be suitable for synaptic applications, the oxide-based resistive random access memory (OxRAM) technology stands out when analog conductivity changes are required [1,2,3,4,5,6,7].

### 1.1. Spike-Timing Dependent Plasticity (STDP) in Memristive Electronic Synapses

The synaptic weight updating process is therefore the basis for the application of any learning algorithm in a neural network, and is related to the capability of the synapse to adapt its conductivity through experience, namely its property of plasticity. In the case of electronic synapses, this feature involves the modulation of the conductivity (G) of an electronic device, where changes (ΔG) can be induced by applying the appropriate voltage drop between its two terminals (Figure 2b). These updates in the conductivity of the device are applied according to the recent activity of the neurons it connects. For instance, temporal correlations and causality between the recent activity of the input and output neurons can determine the magnitude and direction of the relative synaptic weight change, ΔG/G. The so-called spike-timing dependent plasticity (STDP) has been reported in biological systems [8,9,10,11,12,13,14,15,16], and is a popular bio-inspired learning rule implemented in artificial neural networks and computational neuroscience [10,11,12,13,14,15], where ΔG/G is described as a function of the time delay Δt between the pre (input) and post-synaptic (output) neurons spike firing, respectively (Figure 2c). The nature of the synaptic change is what depends on the causality between the input and output neurons activations.

In order to demonstrate the plasticity property of memristive devices, the input and output activities are assumed to be in the form of voltage pulses, and the significant change in ΔG/G occurs when these pulses meet at the terminals of the synaptic device, overlapping in time, causing a significant voltage drop (Figure 2b). In this case, the STDP function (Figure 2c) can be tuned by changing the shape of the input and output neuron pulses [12,13,14,15]. The most popular shape of the STDP, resembling the one reported in a biological culture by Bi and Poo [16] (Figure 2c shows the average of the experimental data), has already been reported in many electronic devices [12,13,17,18,19,20,21]. However, the possibility to tune the STDP function shape, concerning the electronic synapse electrical characteristics, is often skipped. Variety in STDP functions appears in biological synapses [8,12,13,14,15]. This variety extends the application of the STDP as a local learning rule in artificial intelligence learning schemes [12], especially in those based on unsupervised techniques.

### 1.2. Unsupervised Learning and Self-Organizing Neural Networks

Unsupervised learning involves a methodology where the training stage does not require the calculation of any error made by the system for a certain input, in order to improve its performance. That is, both user and system are not meant to know the actual solution of the problem entered to the network, nor detailed information about the input dataset properties, in contraposition with supervised learning techniques. Unsupervised learning implementation would be beneficial for neuromorphic architectures, since on the one hand, it does not rely on the error computation and correction as the supervised learning techniques do, so extra circuitry specialized for this purpose could be avoided. Furthermore, unsupervised learning models, such as the above mentioned STDP learning rule, are considered to be biologically plausible. By reverse engineering simple and primitive biological nervous systems as a first approach, the neuromorphic community would take advantage and inspiration because of the simplicity of their design, compared to the ones found in artificial deep learning neural networks, which present an extremely high density of synapses, neural layers and complex pathways and dynamics. Applications of unsupervised learning algorithms are related to classification, symbolic representation, and associative tasks, usually by extracting the relevant statistical features of the input dataset. Examples of bio-inspired unsupervised learning implementations based on memristive devices for image recognition tasks can be found in [17,18,19,20,21]. However, the hardware-based implementation of other unsupervised learning applications remains unexplored.

**Figure 3:**
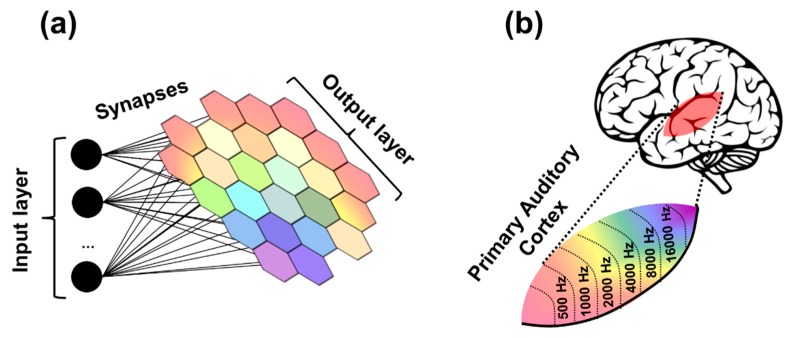
(**a**) Example of a self-organizing map. (**b**) An example of a topological map in the human brain, corresponding to the tonotopic map of the primary auditory cortex.

A particular example of bio-inspired unsupervised learning is the self-organizing map (SOM), also called Kohonen network [22]. Applications of SOM extend to financial predictions, medical diagnosis, or data mining, among others [22,23,24]. The aim of this learning algorithm consists in mapping the input dataset onto a regular and usually two-dimensional grid, which corresponds to the output layer, under an unsupervised and competitive learning scheme. A diagram of a Kohonen network is depicted in Figure 3a. In here, the input layer is unidimensional and consists of three nodes (input neurons). The output layer is bidimensional, and each node corresponds to an output neuron. Output neurons can communicate to their immediate neighbors. All of the input nodes have a weighted connection (synapse) with every output node. The weight of the synapse determines how strong an output neuron responds to the activation of a particular input. These neural networks are inspired in the topological maps found in the sensory-processing areas of the brain (Figure 3b), where neurons that respond to similar inputs are spatially located very close. The key of this algorithm consists in evaluating the similarity between the set of weights of an output neuron and the input data, which is fed to the system as a vector. The original software algorithm consists in the sequential execution of the following steps, parting from a network with randomly initialized weights. For randomly chosen input from a particular dataset, the Euclidean distance between the input and the weights of every output neuron must be computed, in order to determine which is the output neuron whose weights are closer to the input. This element is identified as the best matching unit (BMU) and its weights are updated in order to slightly reduce its distance with the input data.

Once trained, these networks present topographical organization such as the one found in sensory processing areas of the brain, such as the tonotopic map found in the primary auditory cortex, in charge of processing sound (Figure 3b). In here, the neurons that respond to similar sound frequencies are grouped in clusters, which appear in a frequency-ordered fashion. In this way, similar inputs activate neurons in the output layer which are found close to each other, whereas dissimilar ones affect distant regions [22,25,26,27]. The output layer neurons in a trained SOM appear organized in clusters, whose relative size and location provides statistical information of its corresponding input data item characteristics. It is actually the presence or absence of an active response of an output neuron cluster, and not so much the exact input–output signal transformation or magnitude of the response, that provides an interpretation of the input information [22,23,24].

Many methods are derived from the SOM algorithm, where the neural system is built with SOMs as basic blocks or layers, such as the multi-layer or hierarchical SOM (HSOM) [22]. In the latter case, the network is constituted by concatenating SOMS in a feed-forward way (cascade), where one SOM layer is trained by receiving as input the outputs of another previous SOM. The advantage of HSOMs is that they require less computational effort than a standard SOM to perform certain tasks or problems that present a hierarchical or thematic structure, and moreover, HSOMs provide a simpler representation of the results, which leads to an easier interpretation because they allow the user to check what clustering has been performed at each level of the hierarchy. 

In this work, we propose an unsupervised hardware adaptation of the SOM algorithm to be implemented in an on-line learning neuromorphic OxRAM-based crossbar array, by means of bio-inspired unsupervised learning methods, being the first of its kind, to the best of our knowledge. There is another work related to the electronic implementation of the SOM algorithm: [28] is also a simulation work, and is based upon the previous calculation of the desired synaptic weight update, hence not being an unsupervised learning algorithm. In contrast, in our work we provide a fully unsupervised learning algorithm, in which the weight updating process relies on the STDP property of the employed memristive devices. For the sake of simplicity, a very simple input dataset is used as an example, for which a color identification task is provided. First of all, a model from a previous work [29] is used to analyze the plasticity property of the tested devices, which is further verified experimentally. A methodology for tuning the STDP function, which is a key element to control the learning process, is proposed. The obtained STDP curves are used as the local learning rules within the adapted SOM algorithm, for which a fundamental application is demonstrated. The learning mechanisms introduced in this work can concatenate multiple SOMs without extra circuitry, providing a step towards the implementation of hardware-based hierarchical computing systems.

## 2. Materials and Methods

### 2.1. Electrical Characterization and Device Modeling

**Figure 4:**
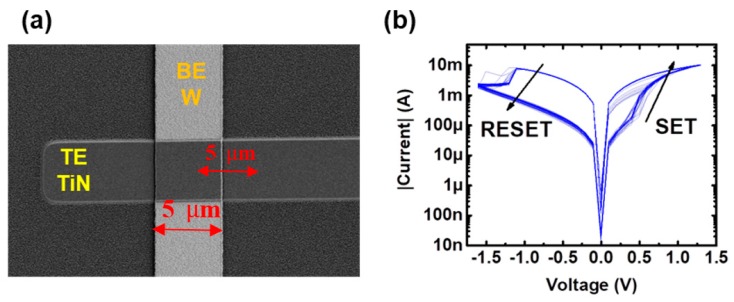
(**a**) SEM image of the tested structure (**b**) Experimental I-V characteristics of the analyzed devices [29]. A voltage limit for the RESET process was set to −1.6V, whereas for the SET process, the conductivity-controlling parameter was the maximum current driving the device (current compliance, I_c_) set by the user.

The devices employed in this study are TiN/Ti-HfO_2_-W metal–insulator–metal (MIM) structures. They were fabricated on silicon wafers either with an oxide isolation scheme or as a single crossbar on a thermally grown 200 nm-thick silicon dioxide. The 10 nm-thick HfO_2_ layer was deposited by atomic layer deposition at 225 °C using TDMAH and H_2_O as precursors, and N_2_ as carrier and purge gas. The top and bottom metal electrodes were deposited by magnetron sputtering and patterned by photolithography. The bottom electrode (BE) consists of a W layer and the top electrode (TE) of TiN on a 10 nm-Ti layer acting as oxygen getter material. The fabricated devices are square cells with an area of 5 × 5 μm^2^. Figure 4a shows a scanning electron microscope (SEM) (IMB-CNM (CSIC), Barcelona, Spain) image of the tested structures, where the TE and BE are indicated. More details on the electrical behavior and fabrication process of these samples can be found in [30,31].

**Figure 5:**
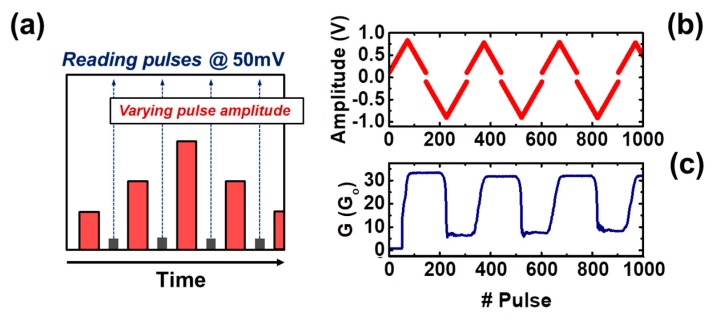
(**a**) Stair-case pulse-programming scheme used in [29] for obtaining the G–V characteristics of a memristive device. (**b**) The pulse amplitude was increased and decreased over time to change the device conductivity. (**c**) Conductivity G as a function of the applied pulses.

In Figure 4b, a few examples of experimental I–V curves are shown, where it can be noted that the tested devices display a bipolar resistive switching behavior, consisting in transitions from high (HRS) to low (LRS) resistance states and vice versa. These transitions are identified as the SET and RESET processes, respectively. The main results of a previous work [30] show that small changes in the conductivity at the low resistance state (LRS) can be induced by means of controlling the maximum current driving the devices during the SET process, proving their plasticity property, and thus indicating that the tested devices are suitable to play the synaptic role in a neuromorphic crossbar-array. In [29], a pulse-programming setup was proposed, with the aim of analyzing in which ways fine changes in the conductivity of the device can be induced by the application of single pulses. The proposed setup allowed obtaining the experimental G–V characteristics of the tested devices, by means of the application of increasing and decreasing amplitude single pulses with a fixed pulse-width over time. Results from [29] are shown in Figure 5, where the pulse amplitude and the conductivity measured after every single applied pulse (in G_o_ units, being G_o_ = 77.5 µS the quantum of conductance unit) are plotted against the number of applied pulses. The conductivity state G was measured after the application of every pulse (Figure 5a, red pulses), by means of applying 50mV (Figure 5b, gray pulses) and reading the current flowing through the device. In the analyzed voltage range, conductivity can take values between ~10 Go and 30 Go.

**Figure 6:**
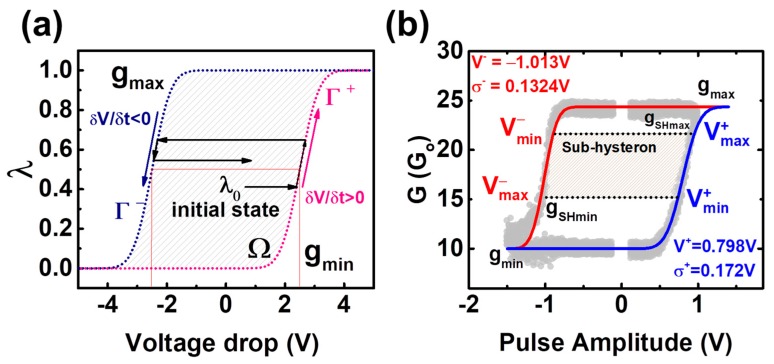
(**a**) Ideal hysteron function of a non-linear memristive device [31]. (**b**) Examples of experimental (gray dots) and an example of a fitted particular case (blue and red lines) G–V characteristics. The fitting parameters V^±^ and σ^±^ are also indicated at the top left and bottom right parts of the figure.

By means of representing the obtained experimental conductivity as a function of the applied voltage, the experimental the G–V characteristics can be fitted according to the compact model of [32]. In here, the so-called hysteron function is used to describe a time-independent conductivity window as a function of the applied voltage in non-linear memristive devices. An example of an ideal hysteron function of a non-linear memristive device is depicted in Figure 6a. The normalized internal state λ is represented as a function of voltage drop at the memristor. The top and bottom boundaries are identified as the maximum (g_max_) and minimum (g_min_) conductivity states. In order to increase (decrease) the conductivity state of the device, a positive (negative) voltage has to be applied so that λ shifts towards g_max_ (g_min_), describing the Γ^+^ (Γ^−^) trajectories. The pair of logistic ridge functions Γ^+^ and Γ^−^ can be modeled with two cumulative distribution functions (cdf) [29], related to the pulse amplitudes applied to the non-linear memristive device, being V^+^, σ^+^ and V^−^, σ^−^ the average and standard deviation values of the cdf related to Γ^+^ (for dV/dt > 0) and Γ^−^ (dV/dt < 0) curves, respectively. Both of them define the boundaries of the possible conductivities of the device within a range limited by the minimum and maximum conductivity states, g_min_ and g_max_, respectively.

In Figure 6b, examples of the experimental G–V characteristics of the tested device are shown, alongside an example of a fitted curve (continuous lines). In here, a conductivity state sub-space is identified as a sub-hysteron (gray area). The main parameters which allow confining the conductivity of a device within the g_SHmax_ and g_SHmin_ conductivity boundaries as the top and bottom limits of the identified sub-hysteron are V^±^_max_ and V^±^_min_. Asymmetry of the obtained G–V characteristics can be noted by comparing the mean value on the two cdf, V^+^ and V^−^, which were used to fit the experimental data to the logistic ridge functions Γ^+^ and Γ^−^. The obtained time-independent empirical model allows computing the conductivity change of the employed devices when single pulses with varying amplitude are applied, such as the ones required for studying the STDP property of electronic synapses.

### 2.2. STDP as a Learning Rule

**Figure 7:**
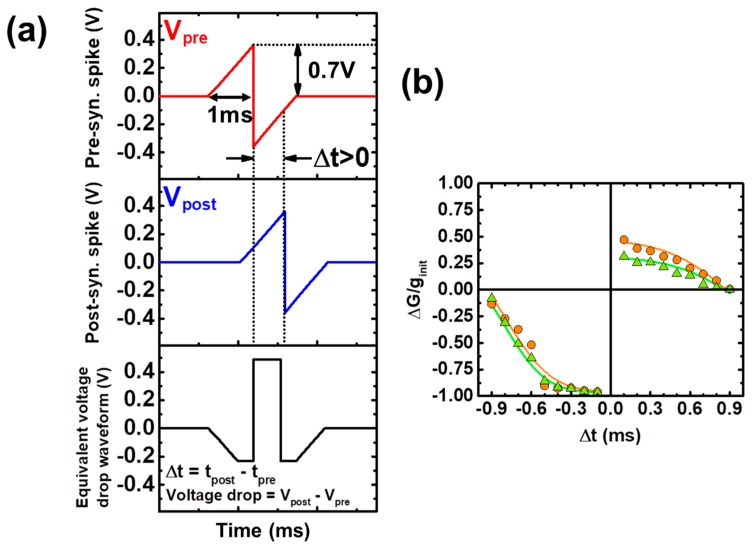
(**a**) Identical pre (red) and post-synaptic (blue) waveforms are applied to the tested samples, which result in an equivalent voltage drop waveform (black) showing a dependence on the time delay Δt. (**b**) Experimental [33] (dots) and modeled (line) STDP functions.

For this application, the experimental STDP windows obtained in [33] were fitted using the above described model. The experimental STDP measurements were obtained by means of applying identical pre and post-synaptic waveforms with a spike width of 1 ms and a maximum voltage of |0.7V_peak_| (Figure 7a), which corresponds to the voltage required to set the conductivity state of the device at g_SHmin_ ~15G_o_ (Figure 6b). Two examples of the experimental and modeled STDP functions are shown in Figure 7b. In here, a bias towards synaptic depression is observed. This biasing is related to the asymmetry observed in the G–V characteristics shown in Figure 6b. Also, saturation of the synaptic weight update is observed for small and negative Δt. This occurs mainly because the voltage drop applied to the device is so large in magnitude, that the reached conductivity state after its application is its lowest value g_min_, so the dependence of Δg with Δt is lost for −0.5 ms < Δt < 0 ms.

**Figure 8:**
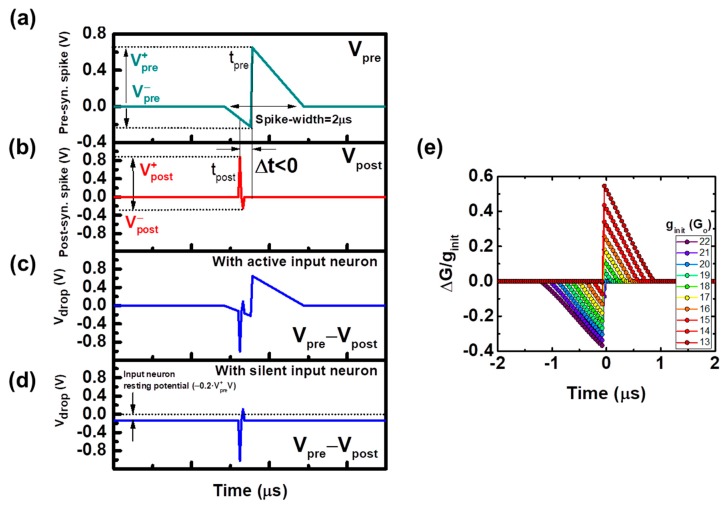
Pair of proposed pre (**a**) and post-synaptic (**b**) waveforms. Resulting voltage drop waveform applied to the sample for an active (**c**) and silent (**d**) pre-synaptic (input) neuron. (**e**) Balanced STDP function. Each curve corresponds to a different initial conductivity state of the same device.

In order to get symmetrical STDP functions, instead of using identical pre and post-synaptic waveforms, we propose using the pair of synaptic pulse shapes shown in Figure 8a (pre) and Figure 8b (post), so the STDP function can be easily tuned in terms of biasing, according to the desired working regime of the employed devices. The resulting equivalent voltage drop applied to the simulated device is depicted in Figure 8c. The maximum and minimum voltage drops at the synaptic device are defined as the V^±^_max_ and V^±^_min_ parameters, respectively (see Figure 6b). By using the proper V^±^_max_ and V^±^_min_ values, a linear operation regime can be achieved (gray area identified as a sub-hysteron in Figure 6b), where the conductivity state can be finely updated according to the STDP rule, and the saturation of ΔG is withdrawn. Moreover, the stochasticity related to the RESET process is avoided. In our case, the following parameters were employed: V_pre_^+^ = 0.7 V, V_pre_^−^ = −0.225 V, V_post_^+^ = 0.875 V and V_post_^−^ = −0.25 V. With these voltages, the conductivity is kept within a sub-hysteron region, in this case ranging from g_SHmin_ = 0.33 (13 G_o_) to g_SHmax_ = 0.8 (22 G_o_).

This procedure allows implementing the balanced STDP functions shown in Figure 8e (simulation), where multiple cases involving different initial conductivity values (g_init_) within the sub-hysteron region are shown. Since there is a dependence on the STDP function shape and g_init_, the symmetry in the induced conductivity changes has to be checked at the normalized conductivity state of g_init_ ~0.5 within the sub-hysteron region, corresponding to g_init_ ~17.5 G_o_ in our case. These results support that symmetrical conductivity changes can be induced by using the proposed pre and post-synaptic waveforms, this symmetry being a key factor for increasing the neural network performance [6].

### 2.3. Self-Organizing Neural Networks Based on OxRAM with Fully-Unsupervised Learning Training

The obtained symmetric STDP function in Figure 8e is used as a local learning rule in a proposed electronic implementation of a unidimensional self-organizing map (SOM). The simulated system consists in a single memristive synaptic layer, which is implemented by an OxRAM-based crossbar array. Input and output neurons share the same structure and functionality, so that the neuron layer roles can be interchanged, and multiple synaptic layers can be concatenated without adding extra circuitry.

**Figure 9:**
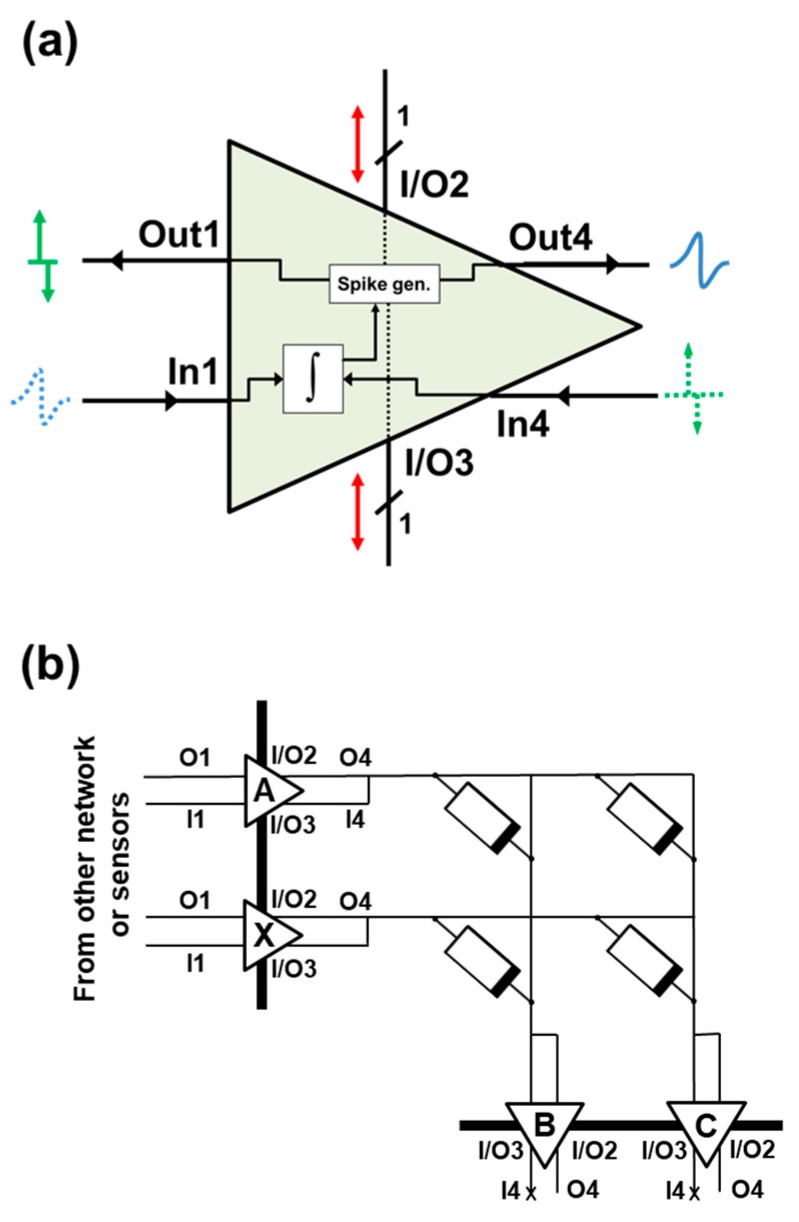
(**a**) Schematic of the proposed electronic neuron, which can play both input and output neuron roles. (**b**) A simplified scheme of the proposed self-organizing neuromorphic network.

The neurons are considered to be integrate-and-fire neurons: the received charge is accumulated, which causes the neuron to depolarize along its membrane (membrane potential), until a certain threshold potential is reached. This process is analogous to a capacitor being charged. Finally, due to this depolarization, the neuron is able to transmit an electrochemical signal towards its synapses, thus communicating with post-synaptic (output) neurons. A schematic of the proposed electronic neuron is shown in Figure 9a. It has six input/output terminals: terminals In1 and In4 receive current signals from the previous and following synaptic arrays, respectively. These signals polarize the neuron and update its accumulated charge, related to the membrane potential. The depolarization is monitored by means of comparing the accumulated charge to a charge threshold, Q_thr_. In the case of an output neuron, when this threshold is reached, the neuron is discharged (its accumulated charge is reset to 0). Then, it triggers a voltage pulse backwards through Out1 and forwards via terminal Out4, towards its synapses. Lastly, I/O2 and I/O3 are communication ports related to the neuron neighbor’s activity signaling, providing communication with the neuron immediate neighbors. For instance, if a neuron fires a pulse, its terminal I/O2 and I/O3 flags will be activated, so its neighbors are warned and will consequently trigger a pulse, which is independent of its actual accumulated charge. When this event occurs, the accumulated charge of the neighbors is also reset. The system depicted in Figure 9b is a simple example of a 2 × 2 crossbar array, showing all of the above mentioned connections. The system consists in two neural layers behaving as the input and output layers. The input and output layers are connected through the 2 × 2 memristive crossbar array, where every intersection corresponds to a weighted connection between an input and an output neuron, provided by a memristor. Adjacent neurons within the neuron layers are connected (black wide line) in order to provide lateral interaction, which is one of the key aspects of the proposed hardware-adapted learning algorithm.

For simplicity, a system with a single synaptic layer is considered in this work. The neuron behavior was included mathematically. Implementations of the designs of electronic neurons based in CMOS technology can be found in [34,35]. In the case of a single synaptic layer system, such as the one depicted in Figure 9b, the input neurons of the system are in charge of triggering voltage pulses through terminal Out4 according to the input dataset (signaled via In1), sourcing or draining current from/to the synaptic layer, and have the integrate function disabled, as well as the neighbor interaction. Output neurons integrate the received current through terminal In1, which corresponds to the summation of each of the input neurons voltage pulse, weighted by its connection weight or device conductivity. These output neurons fire a post-synaptic pulse backwards, as a response to the input neurons activity if their accumulated charge reaches the charge threshold, and also communicate with their immediate neuronal neighbors within the output layer via terminals I/O2 and I/O3. Its activity is measured through Out4. Finally, its terminal In4 is left unconnected. 

A few aspects concerning the learning algorithm are worth to be highlighted: lateral neural neighbor interaction and vertical inhibition within a synaptic column. Lateral neighborhood interaction is one of keys regarding the self-organizing property of the network. According to T. Kohonen in [22], “it is crucial to the formation of ordered maps that the cells doing the learning are not affected independently of each other but as topologically related subsets, on each of which a similar kind of correction is imposed”. This means that when one output neuron receives a signal from a neighbor, which has recently fired a voltage pulse, it is also meant to trigger an identical pulse, both to its own connections with the input layer, and also to its other output neuron neighbor. In other words, the output activity of a particular output neuron propagates through the output neuron layer, leading to the activation of its neighbors. The number of affected neighbors can be defined externally, as well as the shape of the neighborhood interaction function.

**Figure 10:**
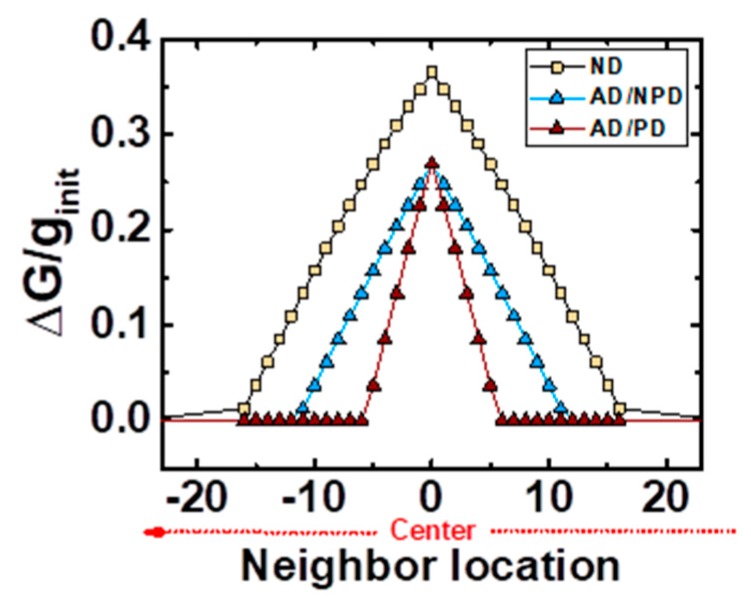
Neighborhood interaction functions based on the STDP rule. The ND curve (yellow squares) shows an example where any delay is considered. AD/NPD curve (blue triangles) consists in a delayed response from the main spiking neuron, but minimum propagation delay. The AD/PD curve is an example of the presence of both delays.

The implementation of a neighborhood interaction function whose amplitude decays laterally is often used in the software versions of the self-organizing networks (Figure 10). This is motivated by both anatomical and physiological evidence of the way neurons in nervous system interact laterally. The most popular choices for this function include a rectangular (abrupt) interaction function, Gaussian (a soft transition) or the so-called Mexican hat function, which consists in a soft transition involving the inhibition of the outermost neurons within the neighborhood. In our case, the decaying amplitude of the neighborhood interaction function is inherent to our system, because of the implementation of the above described STDP function as a local learning rule. Despite the neighbors of the maximally responding output neuron are intended to fire an identical pulse, this pulse will be delayed in comparison with the response of the main responding neuron (center of the neighborhood). With increasing Δt, the induced ΔG/G will also decay with increasing lateral distance, as shown in Figure 10. The radius or number of affected neighbors can be set externally by controlling the time delay: the whole neighborhood activity can be delayed (all delayed, AD), and the propagation delay (PD) between immediate neighbors.

In Figure 10, different neighbor interaction functions are depicted as examples considering different types of delay, where ND states for “not delayed”. The ND curve corresponds to a function where minimum delays are considered: the main firing output neuron B is firing with an accumulative delay AD of one time unit with respect to the last pre-synaptic pulse sent by neuron A, and the PD is also of one time unit. Therefore, the time delay in which a neuron C within the neighborhood fires a pulse after the main responding neuron A has triggered one, as an answer to an input neuron, corresponds to AD + PD·(N+1), being N the number of neurons which separate neurons B and C. In Figure 10, the distance between neurons B and C is none, thus N = 0. The AD/NPD and AD/PD curves present a delay of AD = 5 time units, so that all the conductivity changes in the neighborhood are diminished equally. The difference between these two functions relies on the propagation delay: AD/NPD has the minimum PD, whereas AD/PD has a PD of two time units. As seen in Figure 10, increasing PD results in a narrower function, reducing the number of affected neurons.

Another important aspect is the inhibition of the synapses within the synaptic column of an active neuron. The synaptic column comprises all of its synapses, some of them connecting the neurons with inactive input neurons. For our system, both potentiation of the synapse, relating the firing neuron with the active inputs, and the depression of its synaptic weights which connect it with the inactive inputs, are mandatory to efficiently group or cluster the output neurons, so that a complete correction of the synaptic weights (and thus, of its neighborhood) is performed. This means that if a particular OxRAM conductivity is increased as a result of applying the STDP rule, the other OxRAMs in that synaptic column, connecting the same output neuron with the inactive input neurons, shall be depressed (i.e., their conductivity is decreased). We refer to this process as synaptic inhibition, which leads to an increase of the sensitization of an output neuron to a single input neuron, facilitating clusters specialization to a specific input property. In order to implement this feature electronically, the silent input neurons at a particular time are not actually silent, but rather applying a small and negative voltage through terminal Out4 to their synapses, in analogy with the biological neurons’ resting potential. When an output neuron is firing a pulse backwards, the induced voltage drop at the synapses connecting to a silent input neuron will cause a decrease in their conductivity states. In this case, there is no direct relationship with the STDP rule, since the induced voltage drop at the synapses is not related to any time correlation between the pre and post-synaptic activities. 

**Figure 11:**
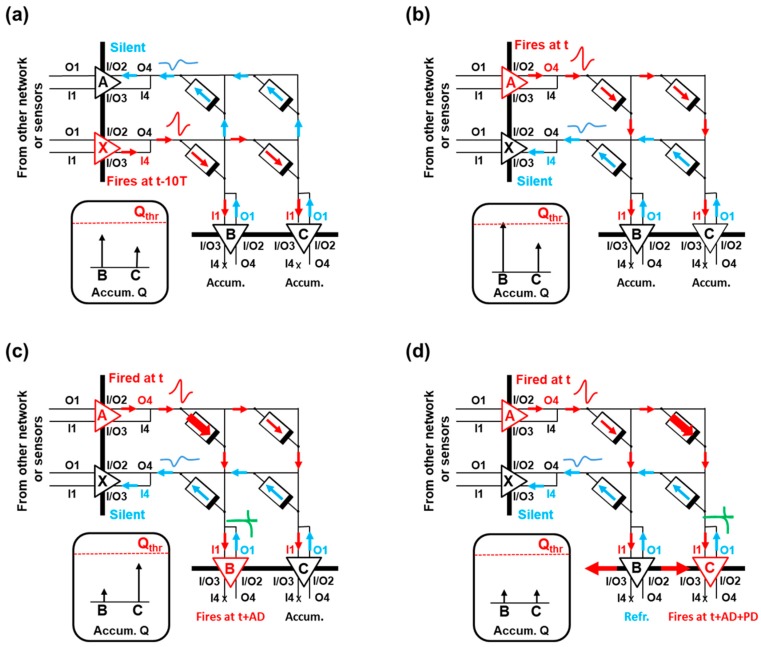
Sketch of the 2 × 2 crossbar array operation. (**a**) Input neuron X fires a pulse through Out4, and input neuron A remains silent. (**b**) Input A fires a pulse, and output neuron B accumulated charge reaches the charge threshold, Q_thr_. (**c**) The accumulated charge of B is reset, and B fires a pulse delayed by AD with respect to the firing time of A. (**d**) Neuron B communicates with its neighbors (only C is depicted). Neuron C triggers a pulse delayed AD + PD with respect to the firing time of A, and its accumulated charge is reset.

A sketch of the operation of the 2 × 2 crossbar array with active and silent neurons, where all of these signals are indicated, is shown in Figure 11. In here, the arrows indicate the current flow in the system. The accumulated charge of the output neurons is also depicted. The input neuron layer consists on neurons A and X, whereas the output neuron layer consists on neurons B and C. In Figure 11a, input neuron X fires a pulse through Out4, and input neuron A remains silent. These signals update the accumulated charge of the output neurons B and C. In Figure 11b, input neuron A fires a pulse, and output neuron B accumulated charge reaches the charge threshold, Q_thr_. In Figure 11c, the accumulated charge of B is reset, and B fires a pulse delayed by a certain delay AD with respect to the firing time of input neuron A. The voltage drop at the synapses within the B column causes a change in their synaptic weights. Then, neuron B communicates with its neighbors (only neuron C is depicted). Finally, in Figure 11d, neuron C triggers a pulse with increased time delay with respect to the firing time of A, AD + PD, and its accumulated charge is reset. Because its pulse presents a larger time delay, the magnitude of the change of its synapses will be smaller, according to the induced STDP function.

Lastly, the methodology suggested for the unsupervised self-organization process to arise is discussed. The synaptic layer is randomly initialized, that is, the conductivity state of each RRAM device is set randomly between the g_SHmin_ and g_SHmax_ values defined previously in Figure 6b. In order to amplify the initial differences between each output neuron synaptic weight values, the threshold potential has to be set large enough, so that the first post-synaptic firing occurs after the presentation of at least 100 pre-synaptic pulses in the case of our electronic synapses. This value takes into account the initial conductivity state values of the employed synaptic devices, and the voltages required to induce the conductivity change according to the STDP function (Figure 8e).

The active input neurons provide current (red arrows) to the output neuron layer, whereas silent input neurons drain current (blue arrows) from the system because of the polarity of its resting potential. In this way, active inputs depolarize the neurons increasing their membrane potential, whereas silent inputs decrease it (Figure 11a,b). The identification of the best matching unit by means of calculating the Euclidean distance of the whole set of synaptic columns is avoided, which simplifies the electronic implementation of the learning algorithm compared to the original Kohonen’s self-organizing learning algorithm, despite a larger number of iterations being required in order to execute this step. On the other hand, if a neuron has recently fired a spike, it will present a refractory period, meaning that it will not be able to fire again after some time, because its accumulated charge has been reset. By doing this, the output neurons which have not fired recently are encouraged to do it. We do not explore the effects of dynamically changing the threshold potential of the output layer. However, a dynamic threshold could improve the performance in terms of convergence time of learning algorithms [36].

**Figure 12:**
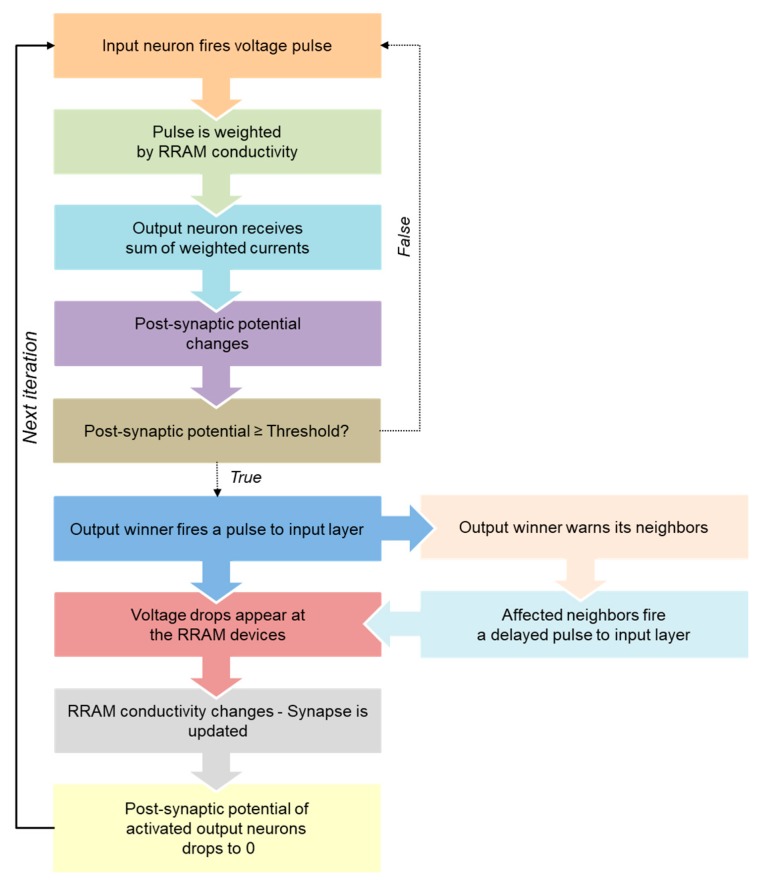
Flow diagram of the self-organizing algorithm based on STDP.

The whole training stage is summarized in the flow diagram depicted in Figure 12. Initially, all of the devices are assumed to have a random conductivity around 15–18G_o_ in our case. The output neurons membrane potentials are also initialized to zero. The input dataset is then fed to the system through the input neurons, which are triggering the pre-synaptic voltage waveform depicted in Figure 8a if active, or applying their resting potential (small negative voltage) to the synaptic array, if silent (as shown in the sketches of Figure 11a,b). The output neurons potentials increase as the output neurons integrate the pulses of the input neurons that they receive, which are weighted by the conductivity of the synaptic devices. That is, the output neurons are receiving a charge whose magnitude is related to the input activity and the weight of the connections between each of them and the input layer. Eventually, one of the output neurons potential will reach the defined charge threshold Q_thr_. At this point, the weight updating process occurs: the output neuron resets its accumulated potential to zero, and triggers the post-synaptic voltage waveform from Figure 8b backwards, affecting its synapses (Figure 11c). The maximum voltage drop given by this post-synaptic voltage pulse and the active input neuron corresponds to the sum of V^+^_pre_ and V^−^_post_ (positive Δt), so this particular synapse is strengthened. On the other hand, the synapses with silent input neurons are depressed, being their voltage drop equal to the sum of V^+^_pre_ and the input neurons resting potential, which is a DC voltage of 0.2·V^+^_pre_ V. Therefore, the induced conductivity change in these synapses has a smaller magnitude in comparison with the one induced to the synapse that connects the winning output neuron with the active input neurons. After the weight updating of the main neuron has been executed, its activity is propagated through the output layer, affecting its immediate neighbors. These other output neurons trigger a voltage pulse with the same amplitude, but with a certain accumulated delay (Figure 11d). That is, the magnitude of the change in the strengthened synapses will be decreasing as the output signal propagates through the output layer, until reaching a non-significant synaptic change, following the neighbor interaction function of Figure 10. The affected neighbors will also reset their output potential to zero. 

In order to reach a convergence state of the map, the maximum synaptic change is diminished by increasing the firing neuron time delay over the iterations. Also, the size of the neighborhood is naturally decreasing over time, since the neighbor firings are consequently delayed. At the end of this training stage, the crossbar weights are organized in clusters, which present overlapped areas. In this way, nearby output neurons will be prompt to react to the same input, whereas distant output neurons will be sensitized to other inputs, as occurs in the software version of the Kohonen map. 

## 3. Application

A fundamental application of the proposed autonomous SOM is shown as an example. In here, a single synaptic layer system of 150 OxRAM synapses is simulated. The synapses are distributed in a 3 × 50 array, 3 being the size of the neuron input layer, and 50 the length of the output neuron layer. The input of the system are the red (R), green (G), and blue (B) color components of a pixel of an image. During the training stage, only one of these components is shown at each time, that is, only one input neuron is firing a pre-synaptic pulse (Figure 8a) with the V_pre_^+^ value as the one shown above (V_pre_^+^ = 0.7 V), i.e., is active at each time. The silent input neurons resting potential is set to a DC voltage of −0.2·V_pre_^+^ = −0.14V. These voltage waveforms are weighted by the synaptic devices conductivities, which are randomly initialized between 15 G_o_ and 18 G_o._ The accumulated charge threshold of the output neurons has to be set in a way that only one output neuron reaches this threshold after a certain time. In the case of the simulated system, the accumulated charge threshold is set to Q_thr_ = 1 mC, so that initially only one output neuron fires a post-synaptic spike. This firing is delayed initially by seven time units (being in our case a time unit t = 0.05 µs, so that initially, AD = 0.35 µs) with respect to the pre-synaptic pulse, so that the maximum relative conductivity change magnitude of a 10% according to the STDP function depicted in Figure 8e. The propagation delay PD is kept constant at five time units = 0.025 µs.

**Figure 13:**
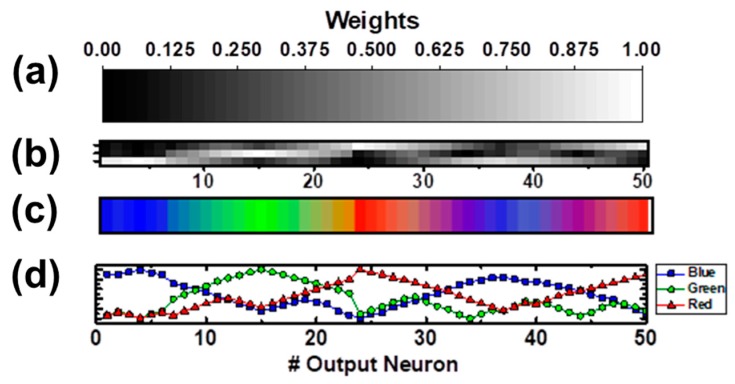
(**a**) Gray-scale used to represent the synaptic weights of the crossbar array. (**b**) 3 × 50 crossbar array displaying the normalized conductivity states of the simulated OxRAM devices after the learning stage. (**c**) Output neuron layer color assignation. The system shows a topographical or spatial organization of the RGB color components. (**d**) Activation response of the output neurons when a red (red line with diamonds), green (green dotted line), or blue (blue line with triangles) color is presented as an input.

Through the iterations, the system is able to self-organize in an autonomous way, without any intervention, being a fully-unsupervised training scheme. After the training stage, the memristors in the column of every neuron within the output layer have a different synaptic weight combination, according to the conductivity states found in the memristors’ column of the output neuron. An example of the obtained topographical pattern is depicted in Figure 13. In particular, Figure 13a displays the gray-scale used to represent the synaptic weights of Figure 13b, which are normalized according to the maximum and minimum conductivity values found in the sub-hysteron region of Figure 6b. The highest conductivity states, depicted in white, correspond to 21G_o_, whereas the lowest ones in black correspond to 13.5 G_o_, being within the defined range of g_SH_ (13–22G_o_). Figure 13b is a representation of the simulated crossbar array after the training, where the synaptic weights are depicted according to the above mentioned gray-scale. The size of this matrix is of 3 × 50 (3 rows and 50 columns), corresponding to the number of input and output neurons, respectively, which are not shown in this representation. It can be seen that, in each of the three rows of the matrix, the synaptic weights increase and decrease gradually. The synapses with the highest synaptic weights of the three rows are located in different regions of the crossbar array, corresponding to the 24th and 50th output neurons in the case of the first row, to the 15th for the second row, and lastly, to the 46th for the third row. The first row of synapses was connected to an input neuron representing the red color component, whereas the second and the third rows were connected to input neurons representing the green and the blue color components, respectively. Then, nearby output neurons appear to have similar colors components assigned, as expected. Hence, groups of output neurons sensitive to one of the primary colors used during the training stage can be identified.

The synaptic weights from every output neuron are related to a RGB coded color, and each of the RGB components is represented by one or two groups of output neurons. The system shows a topographical or spatial organization of the RGB color components. According to Figure 13b, there are output neurons that have a synapse with a large synaptic weight connecting to only one of the three input neurons, whereas their other synapses have a low synaptic weight. This means that these neurons will increase its accumulated charge rapidly, if the input neuron that they are tightly related to shows a strong activity (it fires many pulses in a brief period of time), i.e. these neurons are highly connected to an input neuron, and thus, are highly specialized to a certain color component. Some output neurons, such as the ones found between the 7th and the 13th output neurons, have two synapses with medium synaptic weights, whereas the third one has an extremely low weight. These neurons have a significant relationship with two input neurons, and will respond equally to both of them. If these two input neurons are firing at the same time, because a color consisting of a mixture of green and blue is being used as an input to the system, the output neurons with the two medium-weight synapses will show a stronger response, compared to their response given when only one input neuron is active.

The specialization of the output neurons to a certain input neuron or to a combination of them can be represented by computing the resulting color given by the linear combination of the synaptic weights, relating each of the output neurons to each of the input neurons. The output neuron layer color assignation is represented in Figure 13c, where the color which each of the output neurons is specialized to is depicted. The output neurons’ specialization to a certain color component or its combinations can also be checked by plotting their activation pattern, that is, the change in their accumulated charge due to a certain input activity. Examples of activation patterns of the simulated crossbar caused by single input activity, meaning that only one input neuron is active at a certain time, are shown in Figure 13d, consisting in the increment of the output neurons’ accumulated charge when a red (red line with diamonds), green (green dotted line), or blue (blue line with triangles) color is presented as an input. By means of comparing the output neurons activation as a response of the input data, the system is able to map and classify any combination of the presented colors to the most similar color cluster (i.e., the one showing the highest activation), behaving as a simple self-organizing neural network, such as the software version of the self-organizing map neural network. It is the activation of a particular region of the output neuron layer, corresponding to a certain cluster of output neurons, which gives the information of which input color is being fed to the system. Since the mapping relies on the activation of a group of neurons, redundancy is actually being added to the system. For instance, if one neuron or some synapse is damaged or has an unexpected behavior, the system performance is not going to be affected by it. In a previous work [37], the training reliability of the proposed algorithm was checked. To do so, in [37], different cycle-to-cycle variability levels were considered, and it was proved that the training algorithm presents a significant tolerance to noise and synaptic variability.

The training stage time can be computed in terms of the number of applied pulses and the time scale of the implemented STDP function. The crossbar array after the training shown in Figure 13b was developed within two presentations of the whole input dataset, consisting of 10^6^ pulses of a defined total spike-width T = 2 μs (see Figure 8a), being the time between the input pulses of 10T, which corresponds to a total training time t_T_ = 24 s. The design of the proposed self-organizing map is based on the fact that there is no difference in the electronic design and behavior between the input and output neurons. Because the training scheme is based on hardware-adapted unsupervised learning techniques and the neurons are designed to be able to implement both pre and post-synaptic roles simultaneously (Figure 9a), it is possible to concatenate multiple crossbar arrays, where information flows in a bidirectional manner.

**Figure 14:**
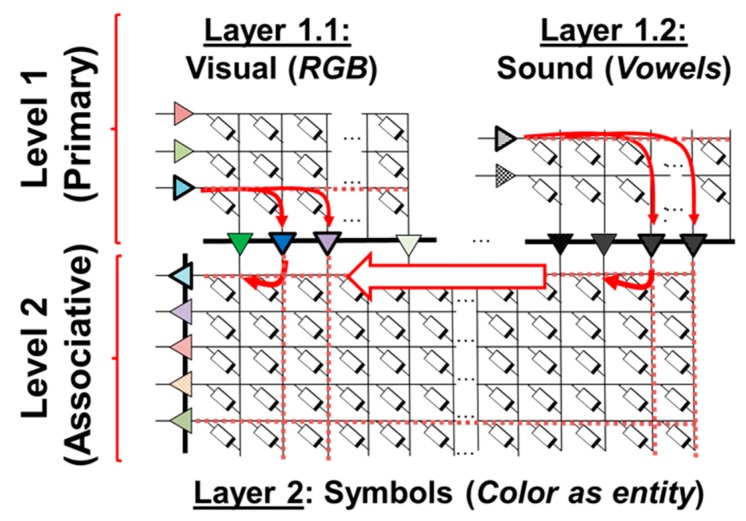
Basic hierarchical computing architecture where the first level (primary) is composed of two memristive synaptic layers, which pre-process the information following the unsupervised algorithm introduced in this work. The output of these layers is fed as the input data of a layer within a higher-order of computation level, where associative learning takes place.

By means of adding computing layers to a self-organizing neural network such as the one presented in this work, hierarchical computation can be achieved. Figure 14 displays an example of a hierarchical SOM system, where the first synaptic layers are constituted by SOMs, such as the color-mapping SOM presented in this work (Layer 1.1), which can also be trained with audio data (Layer 1.2) as to classify the sounds of English vowels. This primary level of the hierarchy (Level 1) pre-processes the information to be fed to higher-order levels, where an associative process between colors and sounds takes place in another SOM, located in Level 2. In other words, the hierarchy permits to develop more complex data structures involving not only the self-organizing property, but also associative learning, which can be summarized as the ability to correlate different memories to the same fact or event [38]. This would represent a step forward towards reproducing complex neural processes and biologically-plausible learning mechanisms in neuromorphic architectures [38].

## 4. Conclusions

Neuromorphic engineering takes inspiration from the biological neural networks learning models, especially when unsupervised techniques are preferred. The most popular learning rule related to unsupervised learning in electronic synapses is the STDP, because it can be easily induced in analog memristive devices, such OxRAM. In this work, a methodology to obtain a symmetrical STDP function in terms of conductivity changes is proposed. It is further applied in the first hardware-adapted version of the self-organizing map (SOM) learning algorithm, which includes other bio-inspired mechanisms in order to achieve topological organization in an autonomous way. This algorithm is performed in a simulated single-layer crossbar array based on the tested devices, for which a fundamental color-mapping application is shown. The introduced system can be potentially used as the basic building block of a multi-layer neuromorphic system, in which hierarchical computing can be achieved without modifying the training algorithm or adding extra circuitry.
